# (−)-Epigallocatechin-3-Gallate Decreases Osteoclastogenesis via Modulation of RANKL and Osteoprotegrin

**DOI:** 10.3390/molecules24010156

**Published:** 2019-01-03

**Authors:** Shih-Tse Chen, Lin Kang, Chau-Zen Wang, Peng-Ju Huang, Hsuan-Ti Huang, Sung-Yen Lin, Shih-Hsiang Chou, Cheng-Chang Lu, Po-Chih Shen, Yi-Shan Lin, Chung-Hwan Chen

**Affiliations:** 1 Department of Psychiatry, National Taiwan University Hospital Hsin-Chu Branch, Hsin Chu 30059, Taiwan; chenshihtse@yahoo.com.tw; 2 Department of Obstetrics and Gynecology, National Cheng Kung University Hospital, College of Medicine, National Cheng Kung University, Tainan 70101, Taiwan; kanglin@mail.ncku.edu.tw; 3 Orthopaedic Research Center, Kaohsiung Medical University, Kaohsiung 80701, Taiwan; czwang@kmu.edu.tw (C.-Z.W.); tony8501031@gmail.com (S.-Y.L.); 327lin@gmail.com (Y.-S.L.); 4 Department of Physiology, College of Medicine, Kaohsiung Medical University, Kaohsiung 80701, Taiwan; 5 Graduate Institute of Medicine, Kaohsiung Medical University, Kaohsiung 80701, Taiwan; 6 Department of Medical Research, Kaohsiung Medical University Hospital, Kaohsiung 80701, Taiwan; 7 Department of Orthopedics, Kaohsiung Medical University Hospital, Kaohsiung Medical University, Kaohsiung 80701, Taiwan; roger01@ms4.hinet.net (P.-J.H.); hthuang@kmu.edu.tw (H.-T.H.); stanelychou@yahoo.com.tw (S.-H.C.); cclu0880330@gmail.com (C.-C.L.); shenporch@gmail.com (P.-C.S.); 8 Departments of Orthopedics, College of Medicine, Kaohsiung Medical University, Kaohsiung 80701, Taiwan; 9 Department of Orthopedics, Kaohsiung Municipal Ta-Tung Hospital, No.68, Zhonghua 3rd Rd., Qianjin Dist., Kaohsiung City 80145, Taiwan

**Keywords:** catechin, EGCG, osteoclast, osteoclastogenesis, RANK/RANKL/OPG, RAW 264.7 cells

## Abstract

Osteoporosis is the second most common epidemiologic disease in the aging population worldwide. Previous studies have found that frequent tea drinkers have higher bone mineral density and less hip fracture. We previously found that (−)-epigallocatechin gallate (EGCG) (20–100 µmol/L) significantly suppressed receptor activator of nuclear factor-kB ligand (RANKL)-induced osteoclastogenesis and pit formation via inhibiting NF-κB transcriptional activity and nuclear transport of NF-κB in RAW 264.7 cells and murine primary bone marrow macrophage cells. The most important regulation in osteoclastogenesis is the receptor activator of nuclear factor-kB/RANKL/osteoprotegrin (RANK/RANKL/OPG) pathway. In this study, we used the coculture of RAW 264.7 cells and the feeder cells, ST2, to evaluate how EGCG regulated the RANK/RANKL/OPG pathway in RAW 264.7 cells and ST2 cells. We found EGCG decreased the RANKL/OPG ratio in both mRNA expression and secretory protein levels and eventually decreased osteoclastogenesis by TRAP (+) stain osteoclasts and TRAP activity at low concentrations—1 and 10 µmol/L—via the RANK/RANKL/OPG pathway. The effective concentration can be easily achieved in daily tea consumption. Taken together, our results implicate that EGCG could be an important nutrient in modulating bone resorption.

## 1. Introduction

The mature, multinucleated osteoclast is the primary cell resorbing bone [1]. Osteoprotegrin (OPG), receptor activator of nuclear factor-kB ligand (RANKL), and macrophage-colony stimulating factor (M-CSF) control the central roles in regulating the proliferation and differentiation of osteoclasts. OPG/RANKL/receptor activator of nuclear factor-kB (RANK) has been set in the current model to study preosteoblastic/stromal cell regulation of osteoclastogenesis [2]. Osteoclasts come from hematopoietic stem cells and their differentiation is controlled by M-CSF, RANKL, and OPG. RANKL can promote osteoclastogenesis in a dose-dependent manner in vitro and can activate pre-existing osteoclasts to rapidly resorb bone in vivo, while OPG reverses the effects of RANKL [3].

Tea from the plant *Camellia sinensis* is a popular drink [4]. More than 80% of green tea polyphenols are catechins (3, 3′, 4′, 5, 7-pentahydroxyflavan). As a potent antioxidant, (−)-epigallocatechin gallate (EGCG) has received the most attention [5,6,7]. Osteoporosis is the second most common epidemiologic disease worldwide. Clinical studies have found that tea consumption can improve bone mineral density and reduce the risk of fractures due to osteoporosis. Habitual tea drinkers have more bone mineral density and less risk of hip fractures [8,9,10]. Despite numerous reports on the effects of tea on the body, osteoclastogenetic effects have rarely been reported [11]. Moreover, the action mechanisms of EGCG on bone remodeling remain unclear.

Our previous study showed that high concentrations of EGCG (20–100 µmol/L) significantly decreased RANKL-induced osteoclastogenesis and function in RAW 264.7 cells and primary bone marrow macrophage cells via inhibiting NF-κB transcriptional activity and nuclear translocation. However, the effect of EGCG on stromal cells with regulation of OPG and RANKL was not reported.

Therefore, we hypothesize that EGCG can inhibit osteoclastogenesis via RANKL/OPG regulation in stromal cells. In this study, we attempted to identity how EGCG regulated osteoclastogenesis via RANKL/OPG modulation.

## 2. Results

### 2.1. MTS Assay

**Figure 1:**
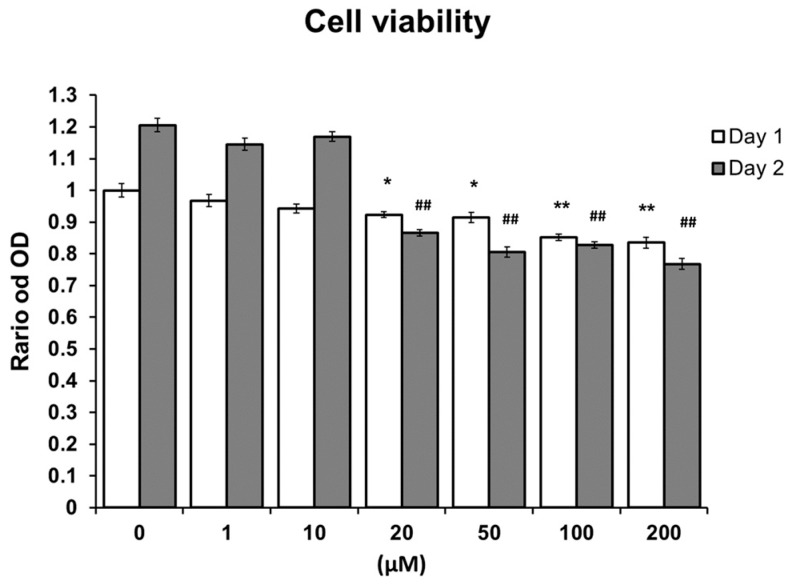
Effects of (−)-epigallocatechin gallate (EGCG) on ST2 cells in MTS. There was no significant change in the MTS assay and cell cycle after EGCG treatment from 1 to 200 µmol/L for 24 and 48 h. With the treatment of EGCG, the viability of ST2 cells was not affected by EGCG at both 1 and 10 µmol/L (both *p* > 0.05). However, decrease via viability of ST2 cells was noted in EGCG concentrations higher than 20 µmol/L. *: *p* < 0.05, **: *p* < 0.01, compared with day 1 0 µM. ##: *p* < 0.01 Compared with day 2 0 µM.

There was no significant change in the MTS assay and cell cycle after EGCG treatment from 1 to 200 µmol/L for 24 and 48 h in ST2 cells (Figure 1). With the treatment of EGCG, the viability of ST2 cells was not affected by EGCG at both 1 and 10 µmol/L (both *p* > 0.05). However, cell viability decreased with EGCG concentration higher than 20 µmol/L. The experiments were repeated at least three times and showed similar effects.

### 2.2. mRNA Expression

**Figure 2:**
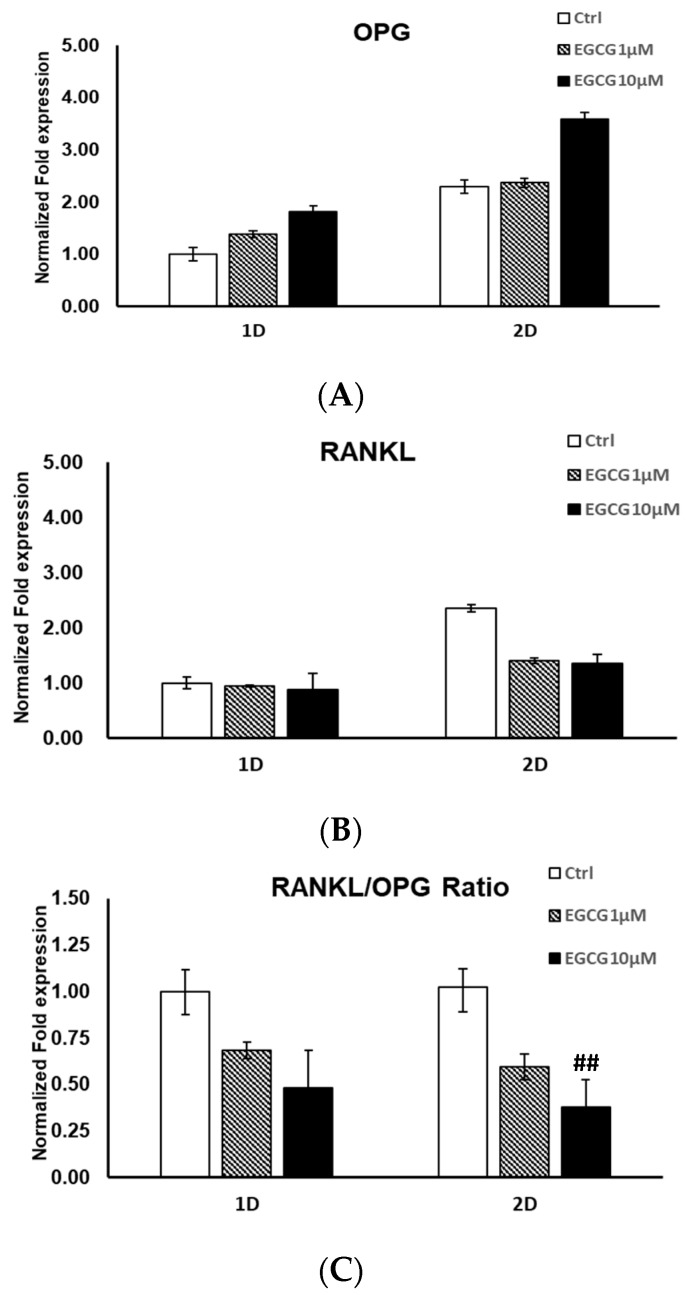
The mRNA expression of osteoprotegrin (OPG) and receptor activator of nuclear factor-kB ligand (RANKL) genes. (**A**) The mRNA expression of OPG increased after EGCG treatment for 24 and 48 h at concentrations of both 1 and 10 µmol/L. (**B**) The mRNA expression of RANKL decreased after EGCG treatment for 24 and 48 h at concentrations of both 1 and 10 µmol/L. However, the difference did not reach statistical significance. (**C**) The ratio of RANKL/OPG decrease at 10 µmol/L after treatment for 24 (*p* = 0.06) and 48 h (*p* = 0.005). ##: *p* < 0.01, compared with day 2 control.

The mRNA expression of OPG increased after EGCG treatment for 24 and 48 h at concentrations of both 1 and 10 µmol/L. The mRNA expression of RANKL decreased after EGCG treatment for 24 and 48 h at concentrations of both 1 and 10 µmol/L. However, the difference did not reach statistical significance. The ratio of RANKL/OPG decreased at 10 µmol/L after treatment for 24 (*p* = 0.06) and 48 h (*p* = 0.005) (Figure 2). The experiments were repeated at least three times and showed similar effects.

### 2.3. Protein Expression by ELISA

**Figure 3:**
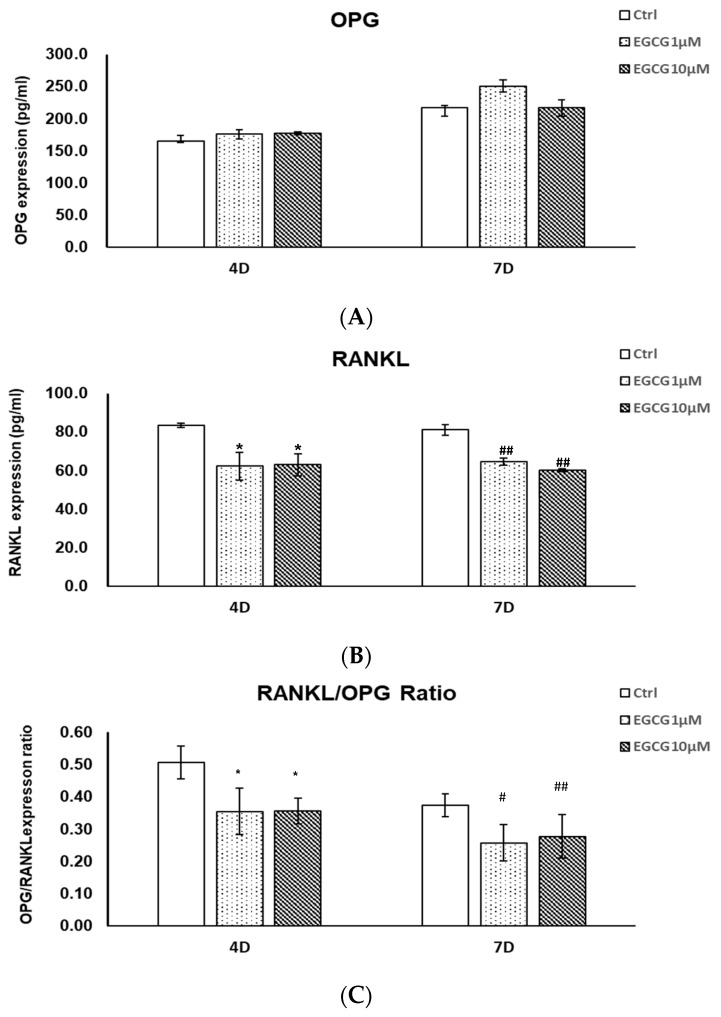
Secretory OPG and RANKL expression by ELISA. There was no significant change in OPG secretion after EGCG treatment at either 1 or 10 µmol/L (**A**). Like mRNA expression, secreted RANKL decreased after EGCG treatment for 4 and 7 days at concentrations of both 1 and 10 µmol/L (**B**). The ratio of RANKL/OPG decreased after EGCG treatment for four (*p* < 0.05) and 7 days (*p* < 0.01) at concentrations of both 1 and 10 µmol/L (**C**). **: *p* < 0.01 compared with day 4 control. ##: *p* < 0.01 compared with day 7 control.

There was no significant change in OPG secretion after EGCG treatment at either 1 or 10 µmol/L. Like mRNA expression, secreted RANKL decreased after EGCG treatment for 4 and 7 days at concentrations of both 1 and 10 µmol/L. The ratio of RANKL/OPG decreased after EGCG treatment for 4 (*p* < 0.05) and 7 days (*p* < 0.01) at concentrations of both 1 and 10 µmol/L (Figure 3).

### 2.4. TRAP Stain and TRAP Activity

**Figure 4:**
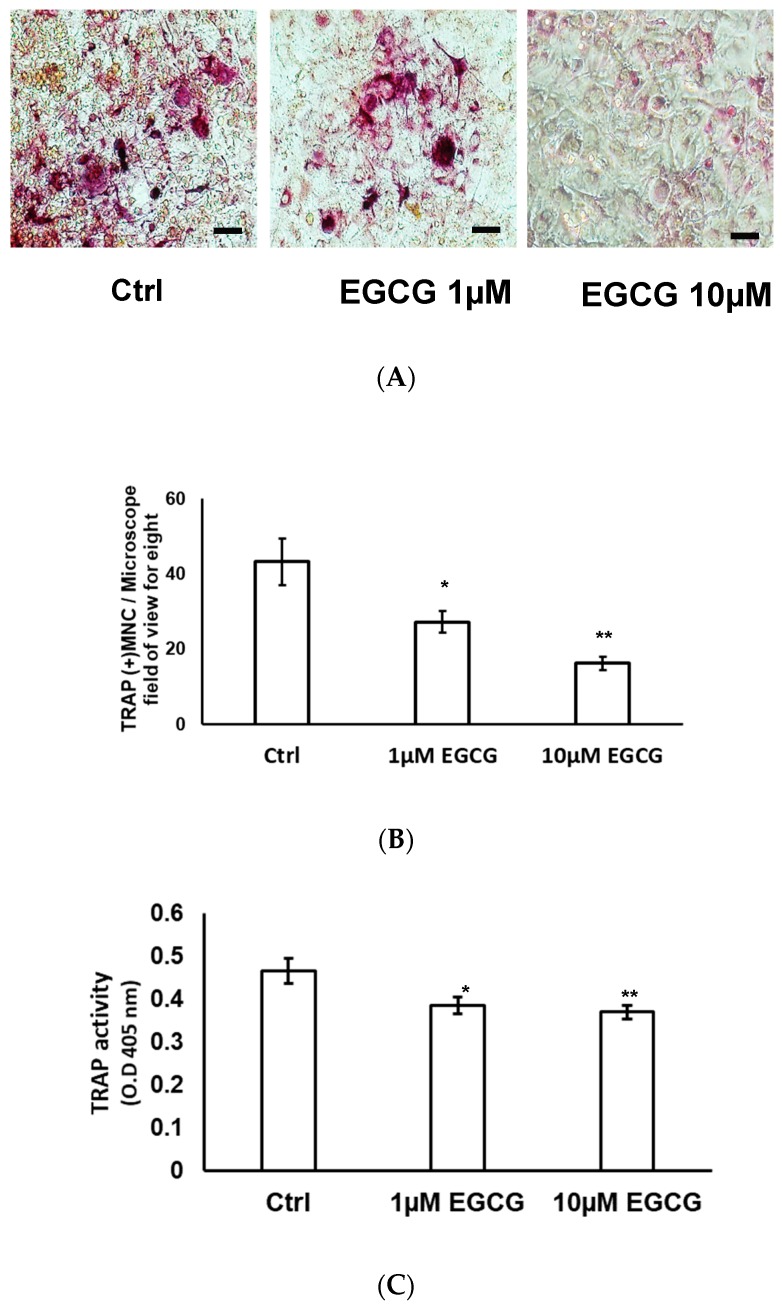
EGCG inhibited osteoclast differentiation of RAW 264.7 cells cocultured with ST2 cells. Coculture cells were seeded in 48-well plates containing α-MEM medium plus 10% FBS, 0.1 μmol/L 1,25(OH)_2_D_3_, and 0.1 μmol/L dexamethasone (DEX). In RAW 264.7 cells, the number of TRAP-positive osteoclasts was reduced by EGCG in a dose-dependent manner (**A**). After quantification, EGCG effectively suppressed the number of multinucleated TRAP-positive cells by 21% at 1 µmol/L (*p* < 0.05) and 91% at 10 µmol/L (*p* < 0.001) (**B**). EGCG at concentrations of 1 and 10 µmol/L dose-dependently reduced the TRAP activity by 17% (*p* < 0.05) and 21% (*p* < 0.01) in RAW 264.7 cells (**C**).

Osteoclast was characterized by the formation of multinucleated cells with a positive TRAP stain. The number of TRAP-positive osteoclasts was reduced by EGCG in a dose-dependent manner in RAW 264.7 cells (Figure 4A). After quantification, EGCG effectively suppressed the number of multinucleated TRAP-positive cells by 21% at 1 µmol/L (*p* < 0.05) and 91% at 10 µmol/L (*p* < 0.001) (Figure 4B). EGCG at concentrations of 1 and 10 µmol/L dose-dependently reduced the TRAP activity by 17% (*p* < 0.05) and 21% (*p* < 0.01) in RAW 264.7 cells (Figure 4C).

## 3. Discussion

We previously found the uncoupling osteogenic effects of EGCG. EGCG can enhance osteogenic differentiation of both murine and human bone marrow mesenchymal stem cells [12,13]. Besides, we also found EGCG can improve bone microarchitecture in ovariectomized rats [14] and healing of femoral bone defects [15]. The most important regulation in osteoclastogenesis is the RANK/RANKL/OPG pathway. In this study, for the first time, we found that EGCG could decrease osteoclastogenesis via RANKL/OPG modulation in murine stromal cells, ST2. EGCG decreased the RANKL/OPG ratio and eventually decreased TRAP (+) stain osteoclasts and TRAP activities.

Reactive oxygen species (ROS) can be detrimental to cellular components such as DNA, protein, and lipids. Cellular metabolites and environmental factors may produce high amounts of ROS and lead to oxidative stress, which perturbs the normal redox balance [4]. Oxidative stress boosts osteoclastogenesis or directly contributes superoxide produced by osteoclasts to bone resorption [16]. A previous study indicated the important roles of oxidative-stress-induced bone loss in osteoporosis [17]. Therefore, antioxidants can decrease oxidative stress, which could potentially ameliorate bone loss in osteoporosis. Green tea polyphenol extracts were found to improve bone loss in aging-induced and aging plus estrogen-deficiency-induced osteopenia animal models [18,19,20,21,22]. Though we found loss of antioxidant activity in EGCG can still enhance mineralization of hBMSCs [13], we also reported that high concentrations of EGCG mitigated osteoclastogenesis through regulation of NF-κB in RAW 264.7 cells at 50–100 µmol/L [11]. Besides, via the Fenton reaction [23] and caspase-3 activation, EGCG increases osteoclast apoptosis [24]. EGCG suppresses bone resorption through a decrease of mitogen-activated protein kinase (MAP kinase) activation [25] or a decrease of interleukin-6 (IL-6) production [26]. EGCG inhibits osteoclastic differentiation through downregulation of the nuclear transcription factor of activated T cell c1 and reduced bone resorption [27,28].

EGCG has been studied extensively as an antioxidant in osteoclast inhibition. The most important regulation in osteoclastogenesis is of the RANK/RANKL/OPG pathway. There is no study about how EGCG regulates the RANK/RANKL/OPG pathway in osteoclastogenesis. We previously found EGCG at high concentrations (20–100 µmol/L) could significantly suppress RANKL-induced osteoclastogenesis in RAW 264.7 cells. There is no effect at a concentration of 5 µmol/L. In this study, we further evaluated the effects of EGCG in cocultures of ST2 and RAW 264.7 cells, which can reflect the effect in regulating the RANK/RANKL/OPG pathway. We found that, even at a low concentration (1 µmol/L), EGCG still could decrease TRAP (+) stain cells and TRAP activity in the ST2 and RAW 264.7 cell coculture system without RANKL supplementation. The effects were more significant at a higher concentration (10 µmol/L). Regulation in the RANK/RANKL/OPG pathway played important roles in EGCG decreasing osteoclastogenesis at low concentrations (1 and 10 µmol/L) via stromal cell modulation.

In conclusion, this study revealed the inhibitory effects of EGCG on osteoclastogenesis at low concentrations via the RANK/RANKL/OPG pathway. One cup of green tea can reach the level of 1 μmol/L EGCG in circulation [5,29]. An oral dose of 1600 mg of EGCG can lead to 7.6 μmol/L in plasma under fasting conditions [30]. In this study, the effective concentration of EGCG to inhibit osteoclastogenesis in the RAW 264.7 cell and ST2 cell coculture via the RANK/RANKL/OPG pathway was 1–10 μmol/L. The effective concentration can be easily achieved in daily tea consumption. Taken together, these results suggest that EGCG could be an important nutrient in the regulation of bone resorption.

## 4. Materials and Methods

### 4.1. Culture of ST2 Cell

Murine bone marrow stromal ST2 cells (American Type Culture Collection [ATCC], Rockville, MD, USA) were cultured in Dulbecco’s modified Eagle’s medium (α-MEM) with 10% FBS and 100 U/mL penicillin/streptomycin (Gibco-BRL, Grand Island, NY, USA) at 37 °C in a humidified atmosphere with 5% CO_2_. After reaching the confluence stage, cells were trypsinized and related at a concentration of 5 × 10^5^ cells/well and incubated in α-MEM with 10% FBS overnight.

### 4.2. Culture of RAW 264.7 Cell

Murine RAW 264.7 cells (ATCC) were cultured in DMEM with 10% FBS and 100 U/mL penicillin/streptomycin (Gibco-BRL, Grand Island, NY, USA) at 37 °C in a humidified atmosphere with 5% CO_2_. After reaching the confluence stage, cells were trypsinized and related at a concentration of 5 × 10^5^ cells/well and incubated in DMEM with 10% FBS overnight.

### 4.3. Coculture System

ST2 cells were plated at a concentration of 10^4^ cells/well in 48-well plates and maintained in α-MEM containing 10% FBS, 0.1 μmol/L 1,25(OH)_2_D_3_, 0.1 μmol/L dexamethasone (DEX), and 1 × 10^3^/well RAW 264.7 cells overnight [31]. Medium was replenished every 2 days. The 1 μmol/L and 10 μmol/L EGCG were treated every day.

### 4.4. Catechin Treatment

Before the experiments, EGCG was dissolved in dimethyl sulfoxide (DMSO) at a concentration of 10 mmol/L and stored at −20 °C for all experiments. The EGCG stock was diluted with culture medium right before treatment. Cells were treated by 1 and 10 µmol/L of EGCG, respectively. Accordingly, the concentration of DMSO was less than 0.1% in the experiments. The cultured medium was changed every 2 days. The experiments were repeated at least three times.

### 4.5. MTS Assay

Briefly, the mitochondria activities of the ST2 cells cultured on wells were detected by the conversion of 3-(4,5-dimethylthiazol-2-yl)-2,5-diphenyltetrazolium bromide (MTS) to formazan [32,33,34]. The quantity of formazan product released into the medium, which was directly proportional to the number of living cells in culture, could be measured by absorbance at 490 nm [35]. Freshly prepared MTS reaction mixture diluted in standard medium at a 1:5 (MTS:medium) volume ratio were added at the indicated time interval to the wells containing the cells and then incubated at 37 °C under 5% CO_2_ for an additional 4 h. After the additional incubation, 100 μL of the converted MTS released into medium from each well was transferred to 96-well plates and the absorbance at 490 nm was recorded with a microplate reader (PathTech, Preston, Australia) using KC junior software [34].

### 4.6. Real-Time PCR

The mRNA level of OPG and RANKL were quantitated by real-time PCR using an iQ5 Real-Time PCR Detection System (Bio-Rad Laboratories, Hercules, CA, USA). In each assay, 1 μg of total RNA was treated with 2U DNase I (Ambion, Carlsbad, CA, USA) and reverse transcribed by the Clontech RT-for-PCR kit (BD Biosciences, San Jose, CA, USA). Real-time PCR reaction mixtures were prepared with iQ SYBR Green Supermix (Bio-Rad Laboratories, Hercules, CA, USA). Melting curve analysis was performed for each reaction to ensure a single peak. Amplicons were visualized with electrophoresis on a 1.4% agarose gel to ensure the presence of a single amplicon. Fold changes (x-fold) in gene expression level were calculated by the 2^−ΔΔct^ method [36]. Real-time PCR was performed with cDNAs from at least three independent experiments. Analysis of variance was performed as in previous studies using Excel 2003 software (Microsoft Corp., Cupertino, CA, USA) [37].

### 4.7. Secretory Protein Expressions by ELISA

OPG was measured in conditioned media from cultures of cells treated with EGCG using the murine OPG/TNFRSF11B Duo Set (R&D Systems, Minneapolis, MN, USA) according to the manufacturer’s instructions. RANKL was measured in conditioned media from cultures of cells treated with EGCG using the murine RANKL (R&D Systems, Minneapolis, MN, USA) according to the manufacturer’s instructions

### 4.8. Differentiation of RAW 264.7 Cells In Vitro by TRAP Staining

Coculture cells were seeded in 48-well plates containing α-MEM medium plus 10% FBS, 0.1 μmol/L 1,25(OH)_2_D_3_, and 0.1 μmol/L DEX. The medium was changed every other day. The medium was removed, and the cell monolayer was washed twice using PBS 5 days after. The cells were fixed in 3.5% formaldehyde for 10 min and washed with distilled water. Cells were incubated at 37 °C in an incubator for 1 h in the reaction mixture of the leukocyte acid phosphatase assay kit (Sigma–Aldrich, St. Louis, MO, USA) as directed by the manufacturer. Cells were washed three times with distilled water, and TRAP-positive multinucleated cells containing three or more nuclei were counted under a light microscope. Total osteoclasts showing TRAP-positive multinucleated cells (with three or more nuclei) were counted under light microscopy by Image-Pro Plus™ [11,38].

### 4.9. Differentiation of RAW 264.7 Cells In Vitro by TRAP activity

Coculture cells were seeded in 48-well plates containing α-MEM medium plus 10% FBS, 0.1 μmol/L, 1,25(OH)2D3, and 0.1 μmol/L DEX. After being cultured for 5 days, RAW-264.7-derived osteoclasts were lysed and incubated for 1 h with a reaction buffer containing paranitrophenylphosphaten (pNPP). The reaction was stopped with 0.3 N NaOH solution 0.3 N, and optical densities (ODs) were read and analyzed by microplate spectrophotometer at 405 nm [11].

### 4.10. Statistical Analysis

All data are presented as mean ± standard error. Comparisons of data were analyzed by one-way ANOVA and multiple comparisons were performed by Scheffe’s post hoc test (SPSS 10.1 Inc., Chicago, IL, USA). *p* < 0.05 was considered statistically significant.

## Running Headline

EGCG decrease osteoclastogenesis via RANKL/OPG.
